# Arterial Hypertension: Individual Therapeutic Approaches—From DNA Sequencing to Gender Differentiation and New Therapeutic Targets

**DOI:** 10.3390/pharmaceutics13060856

**Published:** 2021-06-09

**Authors:** Constantin-Tudor Luca, Simina Crisan, Dragos Cozma, Alina Negru, Mihai-Andrei Lazar, Cristina Vacarescu, Mihai Trofenciuc, Ciprian Rachieru, Laura Maria Craciun, Dan Gaita, Lucian Petrescu, Alexandru Mischie, Stela Iurciuc

**Affiliations:** 1 Department of Cardiology, “Victor Babes” University of Medicine and Pharmacy, 2 Eftimie Murgu Sq., 300041 Timisoara, Romania; costiluca67@yahoo.ro (C.-T.L.); dragoscozma@gmail.com (D.C.); eivanica@yahoo.com (A.N.); mihai88us@yahoo.com (M.-A.L.); vacarescucristina@yahoo.com (C.V.); ciprianrachieru@yahoo.com (C.R.); dr.lauracraciun@gmail.com (L.M.C.); dgaita@cardiologie.ro (D.G.); petrescu_lucian@yahoo.com (L.P.); stela_iurciuc@yahoo.com (S.I.); 2 Institute of Cardiovascular Diseases Timisoara, 13A Gheorghe Adam Street, 300310 Timisoara, Romania; 3 Research Center of the Institute of Cardiovascular Diseases Timisoara, “Victor Babes” University of Medicine and Pharmacy, 2 Eftimie Murgu Sq., 300041 Timisoara, Romania; 4 Department of Cardiology, “Vasile Goldis” Western University of Arad, Bulevardul Revoluției 94, 310025 Arad, Romania; 5 Multidisciplinary Heart Research Center, “Victor Babes” University of Medicine and Pharmacy, 2 Eftimie Murgu Sq., 300041 Timisoara, Romania; 6 Internal Medicine Department, County Emergency Hospital, 5 Gheorghe Dima Street, 300079 Timisoara, Romania; 7 Advanced Research Center in Cardiovascular Pathology and Hemostaseology, “Victor Babes” University of Medicine and Pharmacy, 2 Eftimie Murgu Sq., 300041 Timisoara, Romania; 8 Invasive Cardiology Unit, Centre Hospitalier de Montluçon, 03100 Montluçon, France; alexandru_mischie@yahoo.com; 9 Angiogenesis Research Center, “Victor Babes” University of Medicine and Pharmacy, 2 Eftimie Murgu Sq., 300041 Timisoara, Romania; 10 Multidisciplinary Center for Research, Evaluation, Diagnosis and Therapies in Oral Medicine, “Victor Babes” University of Medicine and Pharmacy, 2 Eftimie Murgu Sq., 300041 Timisoara, Romania

**Keywords:** arterial hypertension, DNA sequencing, gender differentiation of response to antihypertensive treatment, individual therapeutic approach

## Abstract

The aim of this paper is to provide an accurate overview regarding the current recommended approach for antihypertensive treatment. The importance of DNA sequencing in understanding the complex implication of genetics in hypertension could represent an important step in understanding antihypertensive treatment as well as in developing new medical strategies. Despite a pool of data from studies regarding cardiovascular risk factors emphasizing a worse prognosis for female patients rather than male patients, there are also results indicating that women are more likely to be predisposed to the use of antihypertensive medication and less likely to develop uncontrolled hypertension. Moreover, lower systolic blood pressure values are associated with increased cardiovascular risk in women compared to men. The prevalence, awareness and, most importantly, treatment of hypertension is variable in male and female patients, since the mechanisms responsible for this pathology may be different and closely related to gender factors such as the renin–angiotensin system, sympathetic nervous activity, endothelin-1, sex hormones, aldosterone, and the immune system. Thus, gender-related antihypertensive treatment individualization may be a valuable tool in improving female patients’ prognosis.

## 1. Introduction

Despite constant progress in understanding its pathology and associated therapeutic actions by targeting lifestyle changes and novel drug treatment strategies, arterial hypertension currently represents one of the major causes of cardiovascular morbidity and mortality in Europe, with severe potential complications [1,2,3,4].

**Figure 1:**
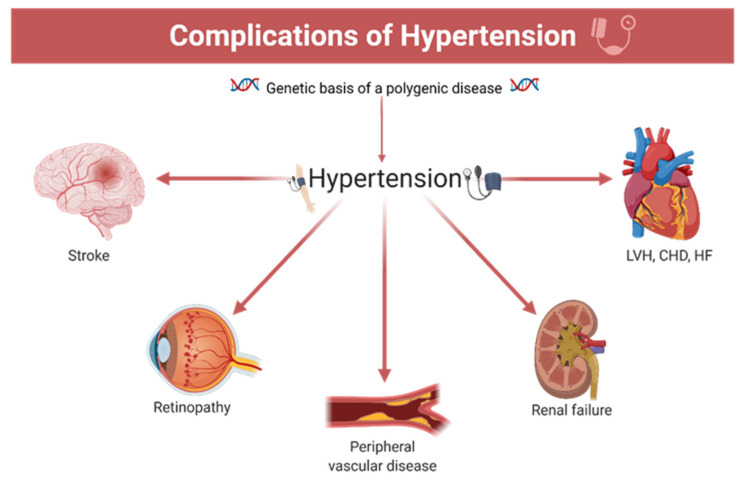
The main complications induced by hypertension (polygenic substrate): LVH—left ventricular hypertrophy; CHD—coronary heart disease; HF—heart failure.

Significant distinct individual variations of responses to antihypertensive therapy suggest that genetic analysis may provide new important data regarding an accurate definition of prognosis and, most significantly, an adequate choice of treatment, therefore preventing potential complications (Figure 1). Data regarding the multifactorial genetic inheritance of essential arterial hypertension have long been considered; however, given the current rise of genetics and genomics, DNA sequencing could represent a step toward specific genetic variation-based therapy in hypertensive patients with uncontrolled blood pressure, despite standard antihypertensive treatment [5].

Seldom isolated, hypertension is often related to a cluster of cardiovascular risk factors. Nevertheless, the prevalence, awareness, and treatment of hypertension is variable in male and female patients. One explanation is that the mechanisms accountable for it may be different and closely related to a gender factor such as the renin–angiotensin system, sympathetic nervous activity, endothelin-1, sex hormones and the immune system. Thus, gender-related antihypertensive treatment individualization may be a valuable tool in improving female patients’ prognosis.

Therefore, in order to achieve better management and adequate control of specific antihypertensive patient categories, the purpose of this overview is to emphasize the importance of the abovementioned issues as they relate to antihypertensive treatment.

## 2. The Importance of Genetics in Arterial Hypertension

Arterial hypertension currently represents the main risk factor responsible for global mortality and burden of disease, with a strong correlation between high blood pressure and cardiovascular disease [6,7].

Thus, a better understanding of the genetic basis of a polygenic disease such as hypertension might bring antihypertensive treatment one step closer to reaching the recommended targeted values of therapy. Likewise, a genetic profile could be helpful in performing individual risk and prognosis stratifications which are based on the identification of specific genetic variants. 

Since an adequate definition of expressed sequences in the human genome represents a useful instrument for defining gene families, proteins and primary patterns of expression in tissues and in specific diseases, a targeted genetic analysis for hypertensive patients would allow for the identification of genes responsible for the disease and an accurate characterization of genomic regions that are linked to arterial hypertension. The study of genetic variants has evolved in the direction of pharmacogenomics: genomic variation inducing individual responses to therapy. This concept can be useful in redefining antihypertensive pharmacological therapeutic actions, as well as for understanding the reason why some individuals respond to standard treatment and others do not. 

Certain factors seem to be connected to the genetic individual variability of a response to antihypertensive treatment: different metabolic profiles and genetic variations of metabolizing enzymes, the genetic variability of sodium sensitivity and proteins from renal tubules (responsible for the regulation of ion transport or variability of response to diuretics) (Table 1).

Concerning etiology, most antihypertensive patients lack an obvious cause and are diagnosed with essential arterial hypertension, a heterogeneous disease with important genetic implications. From this point of view, the Genome Wide Association Study (GWAS) has been an important tool for identifying the genetic implications of essential arterial hypertension [8]. A GWAS provided the opportunity to type, at a large scale, a multitude of Single Nucleotide Polymorphisms (SNP) and, in the setting of large consortia, many studies were published that allowed for the identification of over 100 SNPs that are linked to high levels of blood pressure (see Table 2) [9,10,11,12,13,14].

At first, identifying the cause of essential hypertension remained a difficult task. Using the classic GWAS approach and the “common disease–common variant” hypothesis, genetic variations that could only be responsible for about 3% of essential arterial hypertension genetic causes were highlighted [8]. Thus, the idea that variants too rare to be identified by the GWAS, as well as those which could explain missing information, emerged. The use of new techniques such as systemic genetic approaches, related statistical methods, analyzing intermediate phenotype, transcript, protein and metabolite levels represented an important step in identifying rare genetic causes of essential hypertension. [17] In this field, Exome Chips Based studies and Next generation sequencing are useful tools in underlying rare variants, genes and pathways responsible for inducing essential hypertension. These studies were conducted after the 1000 Genomes Project became available, and they allowed for the development of array-based genotyping platforms (such as Illumina, San Diego, CA, USA) that provided the opportunity to identify a much greater number of nucleotide variabilities in comparison to GWAS [18,19,20,21,22,23,24,25,26,27,28].

## 3. Gender Differentiation of Antihypertensive Treatment

One important inquiry regarding current antihypertensive treatment is whether a gender-related differentiation of this therapeutic approach should be considered in daily medical practice. This judgement is based on several facts. The first is that, despite current gender-neutral recommendations, it is a well-known fact that cardiovascular risks are different in female patients than they are in male patients, with data reporting a higher prevalence for worse risk factor profiles for female patients [29,30,31,32]. Among the factors that may influence this trend are advanced onset age of coronary heart disease in women and delayed medical diagnosis due to a lack of awareness from both health care professionals and female patients concerning coronary heart disease in women. 

Data from the EUROASPIRE V European survey established that, related to cardiovascular risk factors control, women were less likely to perform an adequate level of physical activity and to achieve targeted values for glycated hemoglobin (HbA1c) and low-density lipoprotein cholesterol (LDL-C). Additionally, female patients were more likely to be characterized by obesity, non-smoking and with a medical history of diabetes [30,31,32]. These data are consistent with findings from other studies: the VIRGO (Variation in Recovery: Role of Gender on Outcomes of Young AMI Patients) study included more than 3,500 acute coronary syndrome patients and revealed a higher prevalence for diabetes and obesity in women, and the SWEDEHEART (Swedish Web System for Enhancement and Development of Evidence-Based Care in the Heart Disease Evaluated According to Recommended Therapies) registry evaluated 51,620 coronary patients who reported gender differences regarding blood pressure control and LDL-C targets, with a worsening prognosis for women [33,34]. Similar results were demonstrated by the REACH Registry after analyzing patients with documented arterial disease; analysis revealed that women were more likely to be obese and have uncontrolled cholesterol levels [35]. 

Beyond cardiovascular risk profiles, a gender-related individualization of antihypertensive therapeutic approaches is equally significant. Vynckier et al. reported that, related to antihypertensive treatment, no gender differences were observed for treated hypertension (46.3% vs. 46.6%; *p* = 0.43). Additionally, their results established that women were less likely to have uncontrolled hypertension (30.0% vs. 23.5%; *p* = 0.004), even though they were also more likely to use antihypertensive medication [30]. 

All these data underline the fact that the prevalence, awareness and, most importantly, the treatment of hypertension is variable among male and female patients. 

One other possible clarification is represented by the fact that the mechanisms responsible for this pathology may be diverse and closely related to a gender factor, such as the renin–angiotensin system, sympathetic nervous activity, endothelin-1, sex hormones, and the immune system. Additionally, particular attention should be paid to the key role of aldosterone in influencing cardiovascular risk and immune system activation, with data emphasizing that the efficacy of mineralocorticoid receptor antagonists depends on aldosterone values, indicating a genetic difference in sensitivity to aldosterone [36]. In addition, Te Riet et al. demonstrate that high aldosterone levels in combination with high salt intake may contribute to increased endothelial dysfunction and lead to a rise blood pressure, independent of the renal effects of aldosterone [37].

**Figure 2:**
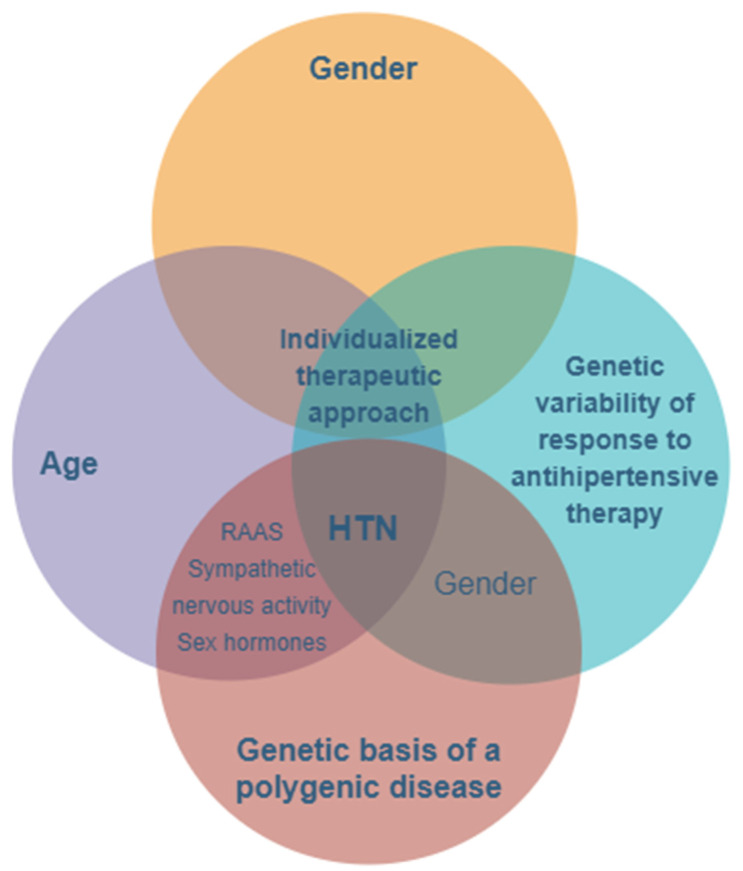
The main features and key factors for personalizing treatment in hypertension with a genetic- and gender-based substrate. HTN—hypertension; RAAS—renin–angiotensin–aldosterone system.

The prevalence of hypertension is higher in men compared to women, at least in young patients under the age of 50. Beyond this age, the prevalence of hypertension is greater among women, with menopause and hormonal imbalances, as well as the activation of the renin–angiotensin system (RAS) and sympathetic and immune systems, contributing to increased blood pressure values [38,39,40,41] (Figure 2).

Last but not least, a recent study by Hongwei et al. indicates that related to specific blood pressure values, lower values of systolic blood pressure are associated with increased cardiovascular risk in women compared to men. They add that the risk of acute coronary syndromes for women with values of systolic blood pressure (SBP) between 110 and 119 mm Hg was comparable to the myocardial infarction risk for men with SBP ≥160 mm Hg [42].

## 4. The Past, the Present, and the Future Analysis 

Recent discoveries in “classical” pathogenic pathways as well as the identification of new pathways are as follows: [43]

- Confirmation of the crucial role of the kidneys in the regulation of blood pressure (BP) and the pathogenesis of hypertension by the intervention of AT1A receptors for angiotensinogen in the proximal convoluted tubules in the regulation of “pressure natriuresis”, as well as by stimulating intrarenal sympathetic nerve activity associated with increased sodium reabsorption and hypertension;
- Deciphering the molecular mechanisms involved in peripheral vascular resistance, especially the signaling pathways activated by the receptors of hormonal mediators coupled with G proteins involved in the regulation of vascular tone and BP;
- Discovering the role of interstitial tissue in the skin as a “dynamic reservoir” of sodium, which buffers the impact of sodium accumulation on intravascular volume and BP;
- Identifying the important role of inflammation and the immune system in the development of hypertension, which also becomes an autoimmune disease (endothelial immunogens cause the infiltration and activation of T lymphocytes in the vascular adventitia, followed by the release of cytokines that increase BP).

All these discoveries have the following important therapeutic consequences: the blockade of AT1 receptors; renal denervation (using a radiofrequency catheter) in refractory forms of hypertension; the blockade of G protein-mediated signaling pathways; and the development of vaccines for hypertension released by the vascular endothelium or against neurohormonal mediators, such as angiotensin II [43].

Although the complex interactions between BP-regulating systems have been extensively studied in recent decades, their specific role in the production of hypertension is not yet fully elucidated. What is certain is that many molecules encoded by genes and whose expression (increased or decreased) is controlled by genetic or epigenetic mechanisms are involved in these processes. In this context, we will again emphasize the inability of researchers to establish the causes of hypertension in each patient, including their genetic vulnerabilities, which is a major obstacle to the development of more personalized and effective clinical management. 

The existence of a genetic component in the production of hypertension has been suggested by the increased family incidence of the disease (even when different members of the same family live in different environments, with varying risk factors), as well as its increased frequency in men and certain ethnic groups, such as in the African American population. For many years, the inheritance of essential hypertension has been a subject with two following hypotheses: [44]

- Platt (1947) measured BP in normotensive and hypertensive people as well as in their relatives, finding a bimodal distribution of BP (patients being a distinct subpopulation compared to normotensive), which led him to state that hypertension is a Mendelian monogenic disease caused by a single mutation
- Pickerring (1959) showed that BP has a quantitative, complex character with a continuous distribution (Gaussian) and polygenic determinism; hypertension (defined by systolic BP values ≥ 140 mm Hg) is only the upper portion (+2.5 standard deviations) of the continuous BP distribution curve. Over time, the Pickering hypothesis has been confirmed by extensive epidemiological studies, and it is now considered that the vast majority of hypertension cases have a multifactorial origin, being produced by the intervention of several susceptibility genes whose effects are modulated by interactions between different genes (epistaxis) as well as between genes and the environment. However, the Platt hypothesis cannot be completely ruled out, as there are rare forms of hypertension and hypotension that are caused by rare monogenic mutations with high penetrance and significant effects. BP certainly shows a phenotypic and genotypic heterogeneity.

After establishing a multifactorial etiological model, an attempt was made to determine the relative contribution of genes to disease determinism. Several recent studies have estimated that the share of heredity (called heritability) in the etiology of hypertension is between 31% and 68%, and the first-degree relatives of patients with hypertension have a three to four times higher risk of developing the disease compared to the general population. However, environmental factors (diet, lifestyle, stress, smoking, alcohol, etc.) play an important causal role, giving HTA the title of “disease of civilization” [45].

The essential problem of hypertension genetics is the identification of genes and the effects of different allelic variants of these genes in the modulation of BP as well as the elucidation of the genetic mechanisms involved in hypertension. Resolving these elements would allow for a correct and complete understanding of the mechanisms of the disease, the establishment of an “individual pathogenic profile”, and the identification of new therapeutic targets. Despite ample, perfectly justified efforts, this problem is difficult to solve.

The study of rare monogenic syndromes affecting BP has identified mutations with major effects in more than 20 genes that produce changes in the following: renal excretion of sodium and/or potassium (e.g., Bartter and Gitelman syndromes, Gordon syndrome, or Liddle’s syndrome); steroid/aldosterone synthesis (e.g., 17α-hydroxylase deficiency or familial hyperaldosteronism); or the sympathetic system (e.g., paragangliomas) [45]. The analysis of these rare but high-effect mutations contributed to the understanding of BP control mechanisms. However, the vast majority of the genetic contribution to multifactorial hypertension has remained unexplained due to the complexity of the disease and its polygenic nature, which involves several genes that each have small effects.

Thus, in order to establish the exact genetic mechanism in hypertension, one step forward could be an accurate identification of genes and their allelic variants that are responsible for blood pressure modulation; this might establish an individual pathogenic profile and bring to light new therapeutic targets of antihypertensive treatment. 

## 5. Identification of Susceptibility Genes Involved in Multifactorial Hypertension

The identification of susceptibility genes involved in multifactorial hypertension is based on linkage and gene association studies, which aim to establish a significant association of a certain chromosomal region or an allelic variant with the disease and are based on the hypothesis of “common disease–common variant”; this hypothesis starts from the premise that the main alleles of susceptibility can be identified in all patients with the same condition [46].

Chain assays were performed in families of patients based on the assumption that patients (usually affected siblings) will more commonly share a certain allele (of a candidate gene) compared to healthy people in the same family. Association studies are performed in a population to compare the incidence of a certain gene polymorphism with its incidence in a control group; if a specific allele of the polymorphic locus has a significantly higher presence in individuals with the disease compared to those unaffected, then it can be assumed that the allele is involved in the pathogenesis of the disease.

Despite the complexity of the disease and its technical difficulties, several gene variants that confer susceptibility to hypertension were identified in the “pregenomic” period, especially genes encoding various components of the renin–angiotensin system, ion channels, or enzymes involved in the synthesis of aldosterone. The following were identified: the ADD1 gene (α-bringing, hydrostatic pressure change sensor, located on chromosome 4p16); the AGT gene (for angiotensinogen, located on chromosome 1q42-q43); the REN gene (for renin, located on chromosome 1q32); the ACE gene (for the angiotensin converting enzyme located on chromosome 17q23); the AT1R gene (for the angiotensin receptor located on chromosome 3q21); the GJA5 gene (for connexin 40, from the junctional structures of endothelial cells in the efferent arteriole and the juxtaglomerular apparatus) for the beta subunit of the non-voltage-dependent sodium channel type 1, located on the 16p13-p12 chromosome); and the CYP11B2 gene (for aldosterone synthesis, located on chromosome 8q21) [46].

In recent years, “case–control” association studies (based on the “common disease—common variant” hypothesis) have been conducted at the genome-wide level (GWAS), accelerating the decipherment of the genetic architecture of BP and HTN. Using genotyping with “high throughput” techniques (SNPs-type DNA microgrids/microchips), about one million genetic markers covering the entire genome can be investigated in a single scan of the human genome. Genotyping of tens of thousands of patients with the same disease and a control group of healthy individuals allowed for the identification of genomic regions that were significantly associated with the disease; these are regions in which variants of disease susceptibility genes are located. The discovery of the causal gene variant (in the identified chromosomal region) and the determination of the mechanism by which this variant participates in the development of the disease obviously require other studies, such as the following: fine mapping to minimize the size of the region in which the susceptibility gene could be found; analysis of DNA sequences in the determined minimum interval to identify the present gene/genes, each with numerous allelic variants; and functional testing of candidate gene variants to experimentally determine the functional effect of the identified gene and its role in disease pathogenesis [47].

From 2008 to 2011, seven GWAS studies for BP and/or hypertension were performed on large populations of different ethnic origins [43,47]. In total, 41 SNPs significantly associated with BP and/or hypertension were identified. In the chromosomal regions (≈100 Kb) or loci defined by these “signals”, the identification of some causal genes proved to be difficult (see Table 2), and only a few genes (NPPA, NOS3, UMOD) were clearly associated with a particular SNP located in the sequence of a gene. Most SNPs were located in regions rich in genes, and then, the “nearest gene” was taken into account (see table). At other times, SNPs involved “regulatory sequences” (binding sites for transcription factors or microRNA molecules) for more distant and yet-to-be identified risk genes.

## 6. Susceptible Genes and their Function

High blood pressure has been shown to be more difficult to decipher than its significant heritability might have suggested. Many identified susceptibility genes have new functions in known pathogenic pathways or are likely to intervene in new pathogenic pathways. The elucidation of these aspects requires further studies but will certainly lead to improving the etiological diagnosis, establishing an individual profile of the disease and optimizing therapy by finding “customized solutions”. In support of this idea, we will refer, in conclusion, to the pharmacogenomics of antihypertensive drugs.

Despite the existence of many classes of effective drugs and many types of drugs in each class, the rates of BP control among hypertensives are disappointing (below 35%), which is mainly due to their low efficacy in some people. In addition, the therapeutic responses to a drug or a combination of drugs differ greatly between patients, as they are highly variable [48,49]. The solution to this drawback is to individualize therapy based on personal genetic information; in short, the pharmacogenomics of hypertension. To date, polymorphisms have been identified in several genes involved in the action of some drugs, which explain the individual variations of their effects: the ACE gene (encodes the angiotensinogen) and the angiotensin inhibitors; the ADD1 gene (encodes α-aducine, hydrostatic pressure sensor) and thiazide diuretics; the ADRB1 gene (encodes the adrenergic receptor b1) and beta-blockers; or the KCNMB1 gene (encodes the beta subunit of the calcium-activated potassium channel) and anti-calcium or beta-blocker drugs. However, the clinical potential of hypertension pharmacogenomics will need to be clearer and more accurate for practitioners to benefit from a rapid test that provides information on the use of first-line antihypertensive drugs. It is estimated that these goals will be achieved by 2022.

## 7. Discussion

Blood pressure control remains an element of utmost importance in patients with cardiovascular diseases and one of the first actions to be considered for patients already diagnosed with coronary artery disease or with heart failure [50,51,52].

Current guidelines recommend similar antihypertensive therapeutic approaches for both male and female patients without making any gender differentiation concerning targeted blood pressure values or for any other specific choice of antihypertensive agents [53].

Additionally, when considering first-line antihypertensive therapeutic agents, women are more likely to be given diuretics and men are more likely to receive β-blockers, angiotensin-converting enzyme inhibitors (ACEI), and calcium channel blockers (CCB) [54,55,56]. One explanation for these therapeutic predilections could be the fact that related to side effects, women treated with ACEI tented to develop cough more often. Additionally, CCB may be avoided in women because of their greater susceptibility to vasodilatation in comparison to men. All these data emphasize the need to focus on implementing gender-related guideline parameters for antihypertensive treatment. Current data suggest that there still is insufficient evidence-based information regarding female patients, at least when regarding the gender individualization of treatment [32,57]. Thus, guideline recommendations are based on clinical trials, typically with a higher prevalence of male patients [58,59,60]. Such was the case of the SPRINT (The Systolic Blood Pressure Intervention) trial, which was a landmark trial regarding blood pressure goals of antihypertensive treatment and characterized by a low female patient enrollment rate and a short follow-up period [61,62,63].

One another important issue remains the implication of genetics and genes, as well as of different allelic variants of genes in the induction and modulation of arterial hypertension. Thus, the Genome-Wide Association Study (GWAS) and array-based genotyping platforms became important tools for better understanding the genetic implications of essential arterial hypertension, and they represent an important step toward a more accurate idea regarding the pathogenic mechanisms of hypertension. The goal would be to establish an “individual pathogenic profile” and identify new therapeutic targets. 

## 8. Further Directions—Genetic Testing, Advantages, and Limitations in an HTN Setting

Arterial hypertension is genetically intricate, which might clarify why the identification of underlying genes has not been as successful as it has for other diseases.

Essential hypertension is a multifactorial pathology in which genetics and the environment both play important roles. It is estimated that a 30–60% variation in blood pressure between individuals can be attributed to genetic factors. The study of hypertension is especially important in children because, with age, it can be modified under the influence of environmental factors. Numerous studies have shown that elevated BP in childhood increases the risk for adult HTN and metabolic syndrome, even with positive environmental changes and healthy growth (mental, social, and dietary) [64].

Genetic testing has become an increasingly used form of medical investigation, with its most varied indications useful for identifying the risk of developing certain diseases and helping to establish the best therapeutic conduct in some pathologies, such as cancer. More recently, nutrigenetics favors the choice of diet and lifestyle according to an individual genetic profile. Therefore, the benefits of genetic testing are indisputable, but there are still a number of limitations and challenges.

Genetic testing involves the analysis of DNA and chromosomes but also the analysis of proteins or certain metabolites for the detection of genotypes, mutations, phenotypes, or hereditary karyotypes related to a disease.

A key moment in the evolution of genetic testing was the sequencing of the human genome, which was the culmination of an international project that was worked on for 13 years. This colossal scientific achievement has facilitated a solid technological platform for studying the genes associated with certain diseases. Another notable step was the introduction of new sequencing techniques, which offered multiple benefits such as rapid cost reduction, much higher resolution levels, increased availability, and an increased scope of genetic testing, especially for single-gene disorders. With the reduction in costs and the emergence of new methods, there is the possibility of sequencing the entire exome or genome, leaving in second place the targeted testing of genes that are considered by a clinician to correlate with the pathology and phenotype of a given patient’s disease [65].

A promising area for the application of targeted genetic testing to personalized medicine is the forecast of responses and adverse reactions to antihypertensive drugs. The identification of genetic markers of drug response will empower the design of randomized controlled trials in a much smaller series of patients than is currently possible, thus diminishing the costs and times from drug design to clinical use and finally providing patients and doctors with a larger number of tools to combat hypertension, which is one the most central risk factors for cardiovascular disease and pathology [66].

In order to achieve its goal, genetic testing must be chosen and interpreted in the context of genetic consultation and genetic counseling. Since genetic consultation is a specialized and complex medical act, genetic examinations are recommended, although these would depend on a patient’s personal and family history, physical and clinical examination, paraclinical examinations, etc. 

Despite the spectacular evolution of genetics and genomics, many challenges remain today, including technical, interpretive, and ethical difficulties. Some mutations remain unidentified or are difficult to decrypt. There are situations in which a genetic test is negative, despite a patient’s symptoms being suggestive of a particular syndrome. The causes of this contradictory result can be multifactorial, and a negative test does not necessarily exclude genetic disease (X). Another limitation is the so-called VUS (variants of unknown significance), i.e., genetic variations with unclear pathological significance. On the other hand, the confirmation of a diagnosis for a genetic condition does not guarantee the existence of a treatment to improve it. In turn, predictive or presymptomatic tests do not provide certainty about the future development of a disease or its degree of severity.

These uncertainties and limitations can lead to significant emotional implications in patients who want more answers. While, for some patients, the results of genetic tests offer a sense of peace or hope, they can also create feelings of fear, anxiety, guilt, anger, discrimination, etc. Therefore, at all stages of the genetic testing process, the psychological support of patients is crucial [67].

Additionally, one final aspect that is just as important as the rest is understanding the family and patient’s perception of HTN and any underlying disease that may contribute to it is importance in resolving any misconceptions and encouraging their adherence to physician recommendations. In order to achieve therapeutic goals, the family must be provided with an adequate education. This education should include appropriate doses of medication, recommended sodium intake, any dietary changes, exercise expectations, and any other behavioral changes.

## 9. Conclusions

Since arterial hypertension remains one of the most influential cardiovascular factors for morbidity and mortality rates, an extensive attempt to provide the optimal antihypertensive medical approach should be considered an important goal in our cardiological practice. An important step toward improving the rate of precise controlled blood pressure values is through identifying genetic underlying pathways and mechanisms accountable for inducing arterial hypertension, as well by the gender-based individualization of antihypertensive therapy.

## Figures and Tables

**Table 1:** Factors influencing genetic variability of response to antihypertensive therapy.

	Factors Influencing Genetic Variability of Response to Antihypertensive Therapy
**1**	Genetic variations of metabolizing enzymes
**2**	Genetic variability of sodium sensitivity
**3**	Genetic variability of proteins regulating renal tubules ion transport

**Table 2:** Polygenic form of essential arterial hypertension, data from [14].

BP Implication	Genes Involved	References
Systolic BP	ATP2B1, CYP17A1, PLEKHA7, SH2B3	Levy et al., 2009 [9]
Diastolic BP	ATP2B1, CACNB2, CSK-ULK3, SH2B3, TBX3-TBX5, ULK4	Levy et al., 2009 [9]
Systolic or diastolic BP	CYP17A1, CYP1A2, FGF5, SH2B3, MTHFR, c10orf107, PLCD3	Newton-Cheh et al., 2009 [15]
Pulse pressure	CHIC2/PDGFRA, PIK3CG, NOV, ADAMTS8	Wain et al., 2011 [16]
Mean arterial pressure	CHIC2/PDGFRA, PIK3CG, NOV, ADAMTS8	Wain et al., 2011 [16]

BP-blood pressure.

## Data Availability

Not applicable.
